# EUS-guided gastroenterostomy using direct needle-puncture technique

**DOI:** 10.1016/j.vgie.2023.10.014

**Published:** 2023-11-02

**Authors:** Judy A Trieu, Todd H. Baron

**Affiliations:** Division of Gastroenterology and Hepatology, University of North Carolina, Chapel Hill, North Carolina

## Abstract

**Background and Aims:**

EUS-guided gastroenterostomy (EUS-GE) is effective in relieving gastric outlet obstruction. Several techniques used to create EUS-GEs have been described. However, these techniques are dependent on passing a guidewire beyond the obstruction. We describe a direct needle-puncture technique that allows for successful EUS-GE creation without a guidewire.

**Methods:**

The direct antegrade EUS-GE method often involves passing a guidewire and tube beyond the obstruction to distend the small bowel. An oblique echoendoscope is then positioned in the stomach to locate the distended small bowel. An electrocautery-enhanced lumen-apposing metal stent (LAMS) is used to create the anastomosis. However, in cases when neither endoscope nor guidewire can be passed across the obstruction, the direct needle-puncture technique can be used. With the oblique echoendoscope positioned in the stomach, a collapsed loop of small bowel is located adjacent to the gastric wall. A 19-gauge needle is used to puncture the gastric and small bowel wall. The small bowel is distended with a mixture of saline, methylene blue, and contrast via a standard water pump connected to the needle. An antispasmodic is administered, and an electrocautery-enhanced LAMS is then introduced into the working channel to create a gastroenterostomy using the freehand method.

**Results:**

The direct needle-puncture technique was performed in 4 patients for these indications: postsurgical inflammation causing gastric outlet obstruction (case 1), tumor infiltration causing gastric outlet obstruction (cases 2A and 2B), and pancreaticobiliary limb access in a duodenal switch (case 3). The video shows the technique performed in a patient with postsurgical inflammation and a patient with duodenal tumor infiltration.

**Conclusions:**

The direct needle-puncture technique is useful for performing gastroenterostomy when the guidewire cannot be passed beyond the obstruction. It can also be used to gain access to a targeted bowel limb in altered anatomy for diagnostic and therapeutic purposes.

## Introduction

Various techniques to create an EUS-guided gastroenterostomy (EUS-GE) have been described.^1^ The direct antegrade EUS-GE method has been widely adopted with high technical and short and long-term clinical successes.^2^ This would typically involve passing an endoscope or at least a guidewire followed by tube placement (usually a nasobiliary tube) across the obstruction. The small bowel is distended with fluid and located with the echoendoscope positioned in the stomach. An electrocautery-enhanced lumen-apposing metal stent (LAMS) would be used to puncture the gastric and small bowel wall to create the anastomosis.

However, the direct antegrade method as well as those previously described are dependent on the ability to pass at least a guidewire across the obstruction. We report the direct needle-puncture technique that allows for successful EUS-GE creation without a guidewire. The technique has been performed in 4 patients in the following clinical settings, summarized in Table 1.

**Table 1:** Summary of the patients’ demographics, procedural information, and outcomes

	Case 1	Case 2A	Case 2B	Case 3
Age (years)	56	65	62	53
Sex	Male	Male	Male	Female
Indication of procedure	Postsurgical duodenal obstruction	Malignant gastric outlet obstruction	Malignant gastric outlet obstruction	Facilitate ERCP in altered anatomy
Procedure time	1 hour, 8 minutes	46 minutes	58 minutes	3 hours, 56 minutes
Additional same-session procedures	None	EUS-guided fine-needle biopsy	EUS-guided fine-needle biopsy	Attempted EUS-guided hepaticogastrostomy
Intraprocedural antibiotics given	No	No	No	Yes (because of dislodged stent)
Stent used	20 × 10 mm LAMS	20 × 10 mm LAMS	20 × 10 mm LAMS	20 × 10 mm LAMS
Technical success	Yes	Yes	Yes	Yes
Clinical success	Yes	Yes	Yes	Yes

*LAMS*, Lumen-apposing metal stent.

## Case 1: Postsurgical Complete Obstruction

A 56-year-old man with a duodenal GI stromal tumor underwent surgical resection with primary duodenojejunal anastomosis. Postoperatively, he never tolerated oral intake, and a CT scan with oral contrast revealed complete obstruction at the surgical anastomosis. A venting G-tube was placed by interventional radiology. An EGD under fluoroscopy confirmed complete obstruction in the first part of the duodenum, and no contrast would pass when infused through the endoscope. After undergoing a direct needle-puncture EUS-GE, he was able to tolerate oral intake and was discharged after a week. However, he returned to the hospital with a fulminant *Clostridium difficile* infection and subsequently died of septic shock because surgery was not an option (Video 1, available online at www.videogie.org).

## Case 2A: Tumor Obstruction

A 65-year-old man with stage IV cholangiocarcinoma complicated by biliary obstruction presented with nausea and vomiting. Cross-sectional imaging was concerning for gastric outlet obstruction caused by tumor compression. An EUS-guided gastroenterostomy was planned; however, the guidewire could not be passed beyond the pylorus because of significant tumor infiltration. A successful direct needle-puncture EUS-GE was performed, and he was discharged from the hospital the following day. He died within 3 months in hospice care.

## Case 2B: Tumor Obstruction

A 62-year-old man with urothelial carcinoma and testicular cancer presented with nausea and vomiting. He underwent an upper endoscopy revealing extrinsic compression in the second part of the duodenum that could not be traversed. Subsequent cross-sectional imaging demonstrated a mass in the pancreatic head. He underwent an EUS with a fine-needle biopsy and planned gastroenterostomy. After the biopsy, a guidewire could not be passed beyond the obstruction. He underwent successful EUS-GE via the direct needle-puncture technique and was discharged the following day. He continues to do well and tolerates an oral diet (Video 1).

## The Direct Needle-Puncture Technique

**Figure 1:**
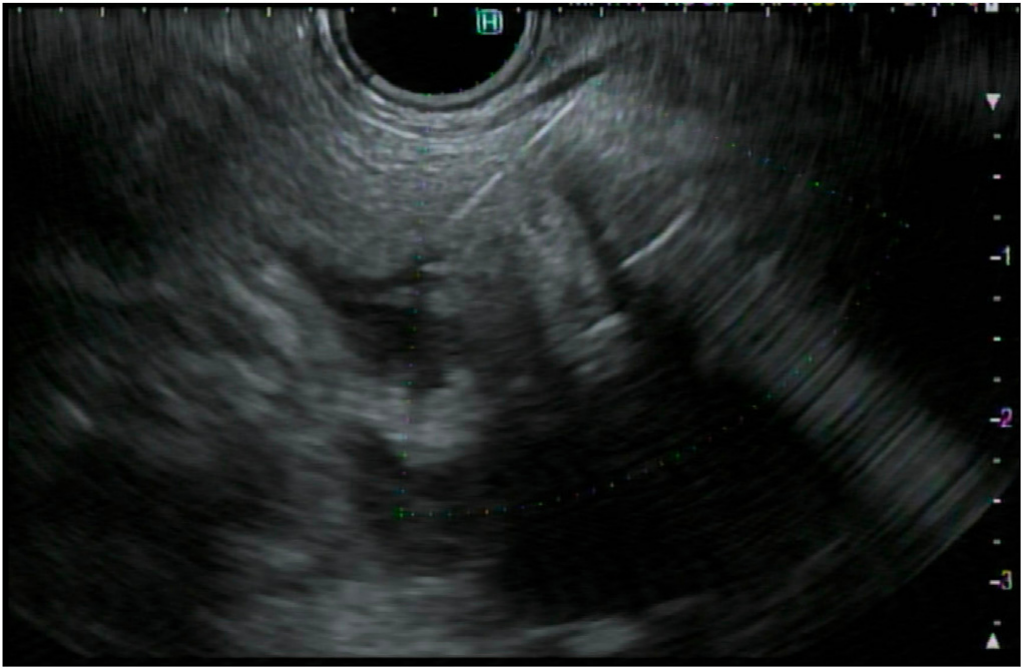
A loop of small bowel was identified under EUS, and a 19-gauge needle was used to puncture through the gastric wall into the small-bowel lumen.

**Figure 2:**
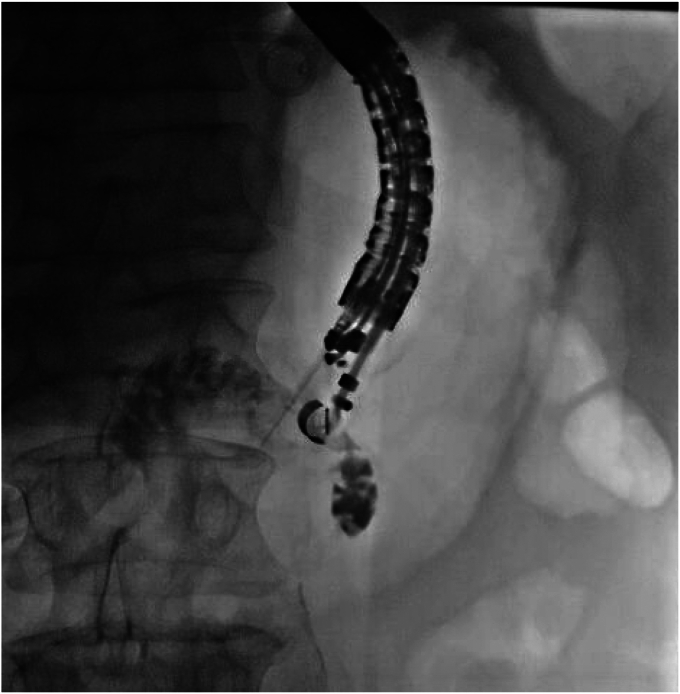
Under fluoroscopy, contrast was injected to ensure that the targeted bowel was distal to the obstruction and ideally the distal duodenum or proximal jejunum.

**Figure 3:**
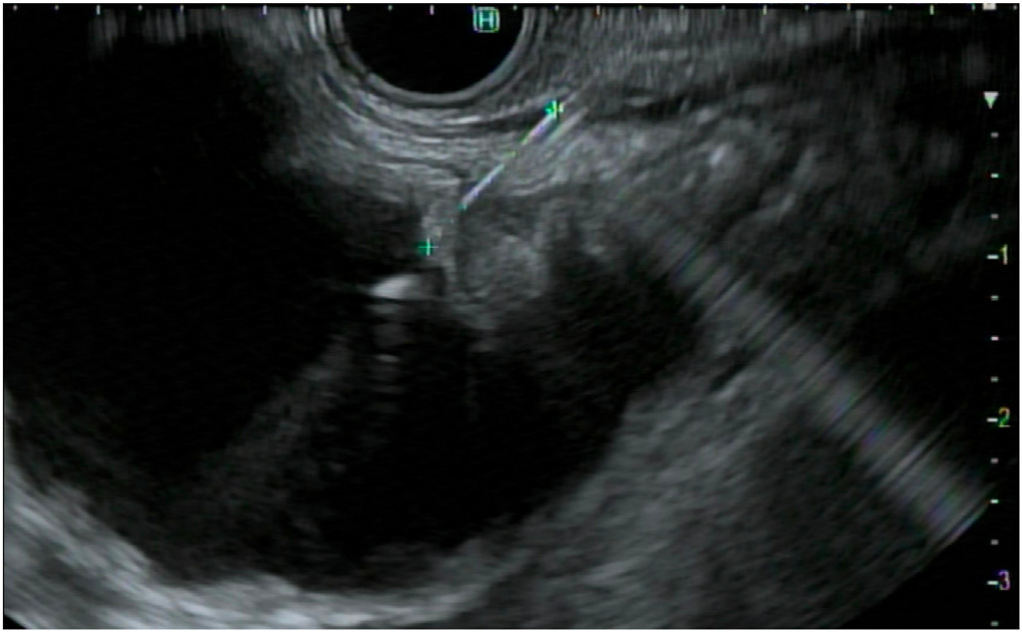
The waterjet was connected to the needle, and the small bowel was distended with saline.

**Figure 4:**
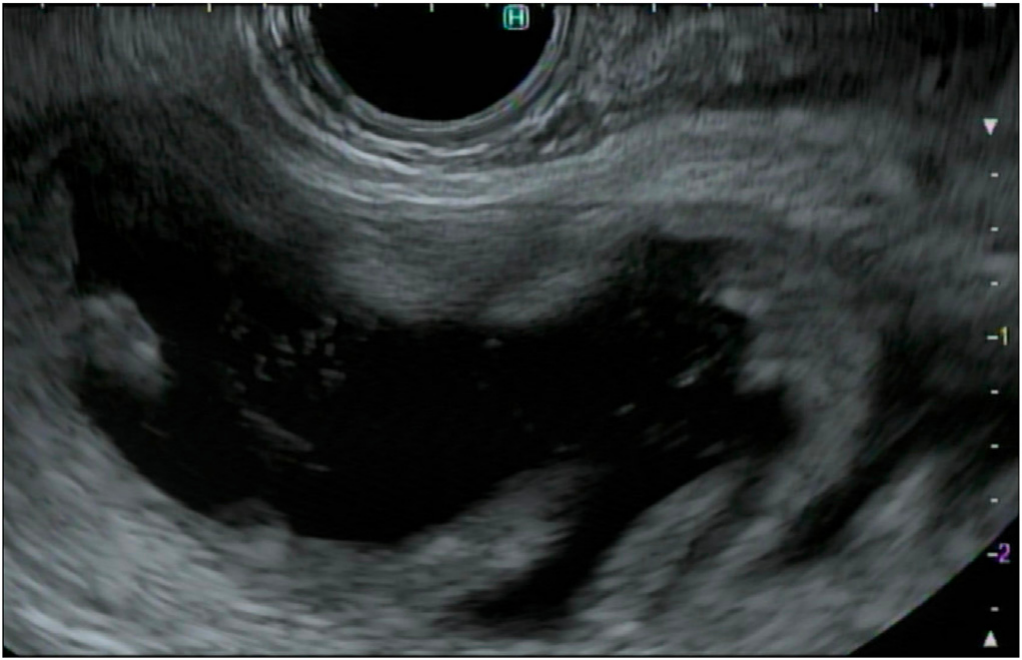
The small bowel was adequately distended to create a large target for placement of an electrocautery-enhanced lumen-apposing metal stent.

**Figure 5:**
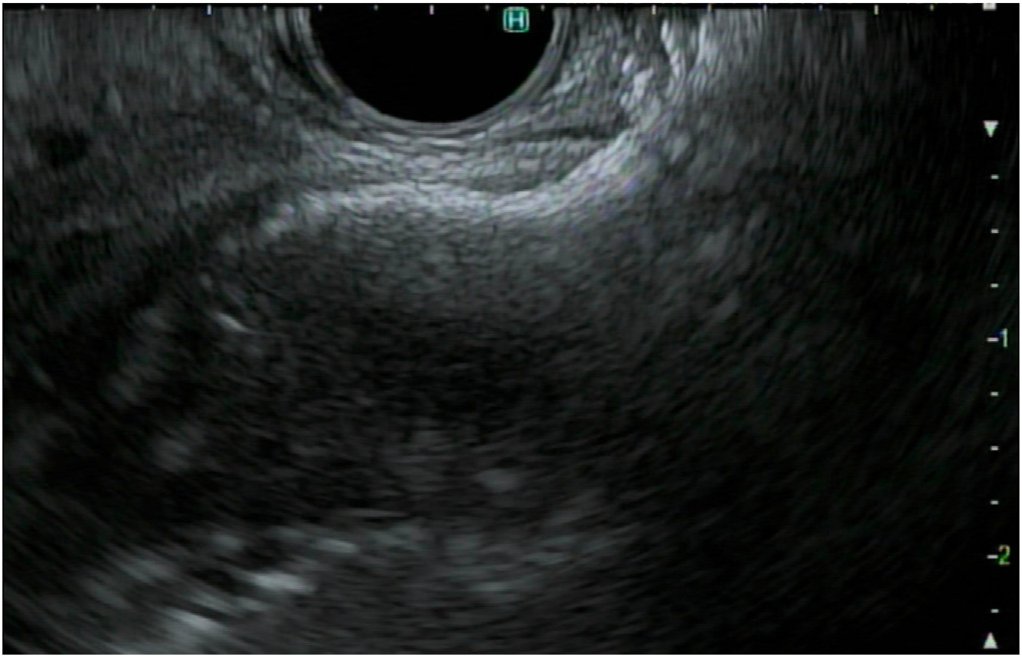
With the direct method, the electrocautery-enhanced lumen-apposing metal stent was successfully deployed with the distal flange seen within the small bowel.

**Figure 6:**
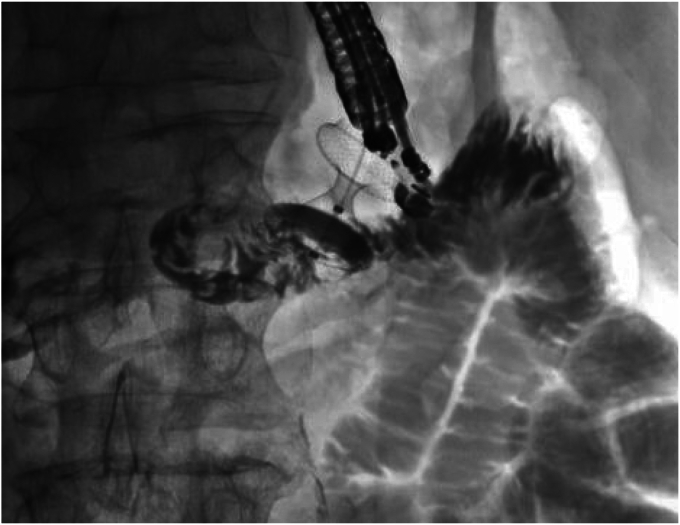
The fully deployed lumen-apposing metal stent was visualized under fluoroscopy with the distal flange in the distal duodenum.

**Figure 7:**
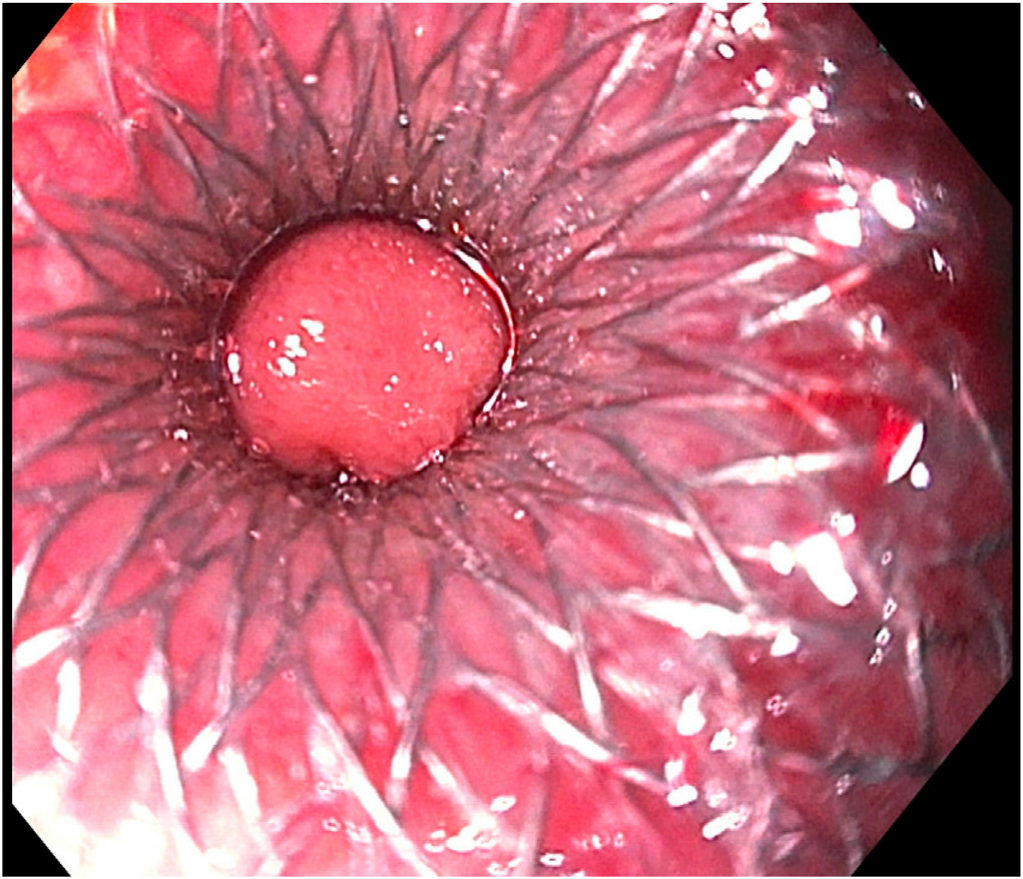
After deployment of the lumen-apposing metal stent, the small bowel was endoscopically visible through the stent.

To create the gastroenterostomy in the aforementioned patients, a loop of small bowel was identified adjacent to the stomach by EUS. A 19-gauge needle was used to puncture the decompressed small bowel (Fig. 1), and contrast was injected to ensure that the target small bowel was distal to the obstruction (Fig. 2). The water pump was then attached to the needle and the small bowel was distended with a mixture of methylene blue, contrast, and saline (Fig. 3). Once the small bowel was adequately distended (Fig. 4), 1 mg of glucagon was given to slow bowel motility. A 20- × 10-mm electrocautery-enhanced LAMS was placed freehand to create the anastomosis under endosonographic and fluoroscopic guidance (Figure 5, Figure 6, Figure 7). Although not used in these described cases because of personal preference and practice, a guidewire may be placed through the 19-gauge needle and then the electrocautery-enhanced LAMS advanced over the wire, particularly in unstable positions. However, the guidewire often pushes the bowel away from the gastric wall. After the procedure, patients were placed on a full liquid diet to allow the stent to fully expand. After 1 day, their diets were advanced to an enteric stent diet (low residue and mechanically soft) indefinitely.

## Case 3: Pancreaticobiliary Access in a Duodenal Switch

This method can also be used in an altered-foregut anatomy when attempting to target and access a specific bowel limb. A 53-year-old woman with a prior bariatric duodenal switch presented with right upper quadrant pain, elevated bilirubin, and mildly dilated intrahepatic biliary ducts. On EUS, the intrahepatic biliary ducts were not dilated enough for an EUS-guided hepaticogastrostomy. To create a duodenoduodenostomy to access the major papilla, a small-bowel limb was identified in the right upper quadrant. This limb was punctured with a 19-gauge needle and confirmed as the pancreaticobiliary limb with contrast under fluoroscopy. The pancreaticobiliary limb was distended with a mixture of saline, contrast, and methylene blue via the water pump, and an electrocautery-enhanced LAMS (20 × 10 mm) was successfully deployed. The major papilla was accessed through the duodenoduodenostomy, and an ERCP was successfully performed with removal of the stones and sludge. After the ERCP, the duodenoduodenostomy LAMS was noted to have dislodged, and a salvage fully covered 18-mm × 6-cm esophageal stent was placed across the anastomosis. She was given antibiotics and was discharged after 4 days. She returned 5 weeks later to exchange both metal stents for two 10F × 4-cm double-pigtail stents to maintain access to the pancreaticobiliary limb. She continued to do well after the procedure.

## Discussion

EUS-guided gastroenterostomy has been an increasing alternative to treat both benign and malignant gastric outlet obstruction. Previously, duodenal stenting was the only option in nonsurgical candidates; however, the rates of stent migration and stent failure/occlusion are high.^3^^,^^4^ Retrospective studies comparing EUS-GE to surgical GE revealed equally high rates of success and efficacy with lower morbidity in the endoscopic group.^5^^,^^6^

EUS-GE had been described to facilitate ERCP in patients with altered-foregut anatomy. These patients had percutaneous transhepatic biliary drains placed with the distal tip near the biliary anastomosis, which was used to distend the small bowel.^7^ This is another alternative if the pancreaticobiliary limb cannot be identified using the direct needle-puncture technique.

Various adverse events have been reported following EUS-guided gastroenterostomy with the most dreaded one being stent misdeployment.^8^^,^^9^ To decrease this risk, glucagon was given prior to puncture of the small intestine with the LAMS in our cases, as opposed to during the distension of the small intestine, which may be the practice of others. Particularly in the direct needle-puncture technique, the inability to continue to distend the small bowel via a nasojejunal tube can result in a less than ideal target, increasing the risk of stent misdeployment. The rates of bleeding and infection are low. Antibiotics are not universally used^1^ and typically are only given at the discretion of the endoscopist. In our practice and in the patients described in this series, antibiotics were not routinely given. An adverse event unique to the direct needle-puncture technique is puncturing and injecting intramurally when targeting a small-bowel limb. In addition, a more distal jejunal limb may be inadvertently used to create the anastomosis, which can result in diarrhea and/or malabsorption.

When the guidewire cannot be passed because of surgical inflammation or an infiltrating tumor across the obstruction or when a specific bowel limb needs to be accessed, the direct needle-puncture technique can be used to successfully create an EUS-guided gastroenterostomy or enteroenterostomy. This technique was described previously.^10^ However, a 22-gauge needle was used to first puncture the targeted small bowel and then exchanged for a 19-gauge needle. Our series shows that this technique using a 19-gauge needle alone can be successful while decreasing the risk of the bowel moving away during device exchange. The direct needle-puncture technique is a viable alternative when traditional EUS-GE methods cannot be performed.

## Disclosure

Dr Baron is a consultant for Cook Endoscopy, Boston Scientific, Olympus, Medtronic, ConMed, and W.L. Gore. Dr Trieu disclosed no financial relationships relevant to this publication.

## Supplementary data

Video 1 In postsurgical anatomy, the EUS-guided needle-puncture technique can be used to identify and distend a loop of small bowel adjacent to the gastric wall, allowing endoscopic creation of a gastroenterostomy.
